# Lipoleiomyoma of the Uterus and Primary Ovarian Leiomyoma in a Postmenopausal Woman: Two Rare Entities in the Same Individual

**DOI:** 10.1155/2015/564846

**Published:** 2015-04-27

**Authors:** Sefa Kelekci, Serenat Eris, Emine Demirel, Serpil Aydogmus, Nese Ekinci

**Affiliations:** ^1^Perinatology Department, Izmir Katip Celebi University Ataturk Training and Research Hospital, Basin Sitesi, Yesilyurt, 35360 İzmir, Turkey; ^2^Gynecology and Obstetrics Department, Izmir Katip Celebi University Ataturk Training and Research Hospital, Basin Sitesi, Yesilyurt, 35360 İzmir, Turkey; ^3^Pathology Department, Izmir Katip Celebi University Ataturk Training and Research Hospital, Basin Sitesi, Yesilyurt, 35360 İzmir, Turkey

## Abstract

Uterine lipoleiomyomas are rare benign tumours that are composed of various mixtures of smooth muscle and mature fat tissue. Leiomyomas, which arise primarily in the ovary, are extremely rare tumours that account for 0.5–1% of all benign ovarian tumours. To the best of our knowledge, we present the first case of an ovarian leiomyoma coexisting with a uterine lipoleiomyoma in the postmenopausal period. A 59-year-old, gravida 4, para 3, postmenopausal woman exhibited pelvic discomfort and increased frequency of micturition. A pelvic examination revealed a solid, tender mass on the left side that could not be clearly separated from the uterus. She underwent a laparotomy with an initial diagnosis of a left ovarian mass. She had previously undergone a total abdominal hysterectomy and bilateral salpingo-oophorectomy. A histopathological examination revealed a uterine lipoleiomyoma, composed of variable amounts of smooth muscle cells and mature adipocytes and a right ovarian leiomyoma composed of interlacing bundles and fascicles of spindle cells. The coexistence of these two rare entities in the same individual may represent a common pathway as a stimulating agent. This case may help to clarify the pathogenesis of these lesions.

## 1. Introduction

Uterine lipoleiomyoma is a rare benign tumour that is composed of various mixtures of smooth muscle and mature fat tissue [1]. The incidence of uterine lipoleiomyomas varies from 0.03% to 0.2% [2]. They are typically found in postmenopausal women ranging from 50 to 70 years of age and they are associated with ordinary leiomyomas [3].

Leiomyomas arise primarily in the ovary but are also an extremely rare tumour, accounting for 0.5–1% of all benign ovarian tumours [4]. The majority of such tumours occur in the reproductive period. Most of them are unilateral and no bilateral cases have been described in patients over the age of 35 [4, 5].

To the best of our knowledge, we present the first case of an ovarian leiomyoma coexisting with a uterine lipoleiomyoma in the postmenopausal period.

## 2. Case Presentation

A 59-year-old, gravida 4, para 3, postmenopausal woman exhibited pelvic discomfort and increased frequency of micturition. Her obstetric and gynaecological history was normal. She experienced menopause at the age of 53 and was not on any hormone replacement therapy. A pelvic examination revealed a solid, tender mass on the left side that could not be clearly separated from the uterus. It was difficult to differentiate whether it originated from the uterus or the ovary.

A pelvic ultrasound revealed a normal-sized uterus and a 10 × 8 × 8 cm heterogeneous mass without vascularity. Her CA 125, CEA, CA 19-9, and AFP values were within normal limits.

She underwent a laparotomy with an initial diagnosis of a left ovarian mass. At laparotomy, her bilateral ovaries and tubes were normal in size but there was an 8 cm mass arising from the posterior fundocorporal region of the uterine wall. The patient had previously undergone a total abdominal hysterectomy and bilateral salpingo-oophorectomy. The postoperative period was uneventful and she was discharged from the hospital 48 hours after the operation.

**Figure 1:**
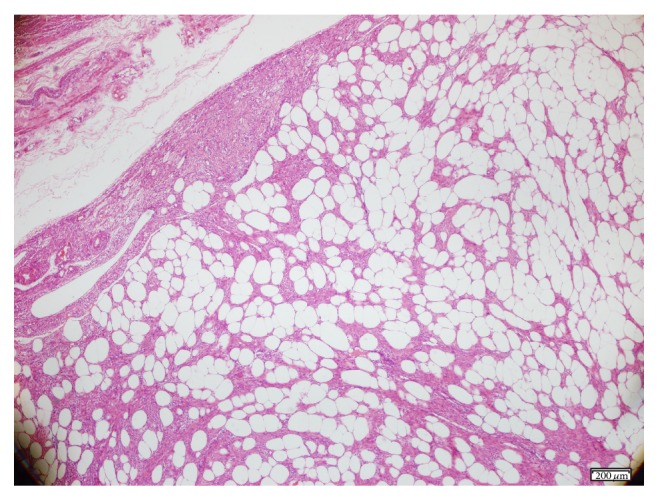
Uterine leiomyoma, H-E ×4.

**Figure 2:**
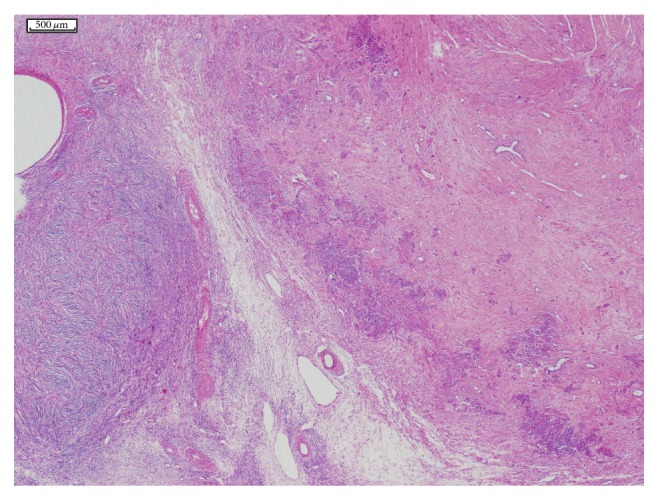
Ovarian leiomyoma with the ovarian capsule on the left, H-E ×2.

**Figure 3:**
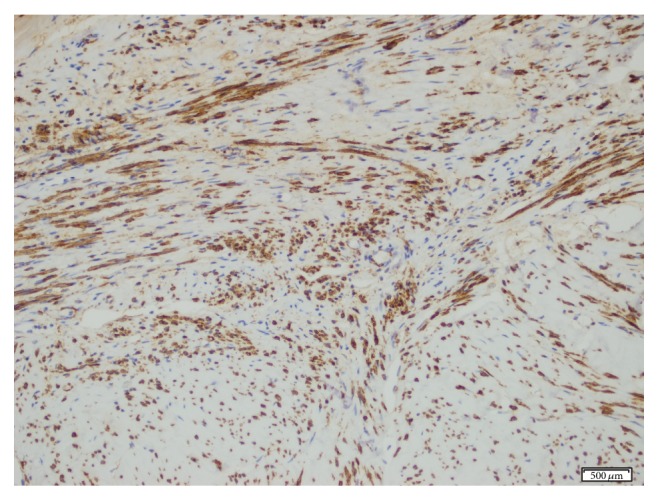
Diffuse positivity with alpha-smooth actin ×10.

A macroscopic examination revealed an 8 cm tumour in the fundocorporal region of the uterine wall with a lipomatous cut surface. There was also a 1.2 cm tumour in the cut surface of the right ovary. A histopathological examination revealed a uterine lipoleiomyoma, composed of variable amounts of smooth muscle cells and mature adipocytes (Figure 1), and a right ovarian leiomyoma composed of interlacing bundles and fascicles of spindle cells (Figure 2). No cellular atypia or significant mitotic activity was seen in either tumour. To confirm the diagnosis of the tumours, the tissue sections were stained with h-caldesmon and alpha-smooth muscle actin, which yielded a positive reaction (Figure 3), and with HMB45, which yielded a negative reaction immunohistochemically.

## 3. Discussion

Lipoleiomyomas are uncommon fatty tumours of the uterus. They are typically found in the uterine corpus intramurally; however, extrauterine locations such as broad ligament, ovary, or peritoneum have been also reported [6]. They are typically found in postmenopausal women; 90% of patients are older than 40 [2].

The clinical manifestations of lipoleiomyomas are identical to those of uterine leiomyomas; the majority of patients are asymptomatic. However, some patients present symptoms such as pelvic pain, abnormal uterine bleeding, constipation, and increased urinary frequency associated with the size of the lesion [1–3].

Lipoleiomyomas are composed of variable amounts of smooth muscle, mature adipocytes, and fibrous tissue. Many theories have been proposed to identify the origin of mature adipocytes, including misplaced embryonic fat cells or stromal mesenchymal cells and lipomatous metaplasia of smooth muscle cells [7]. Leiomyomas, leiomyosarcomas, and ovarian teratomas should be considered in differential diagnoses [1–3].

Primary leiomyomas are one of the rarest solid tumours of the ovary. The majority of these tumours are small and asymptomatic, so they often are discovered incidentally at surgery or at autopsy [4, 5]. Clinical presentations such as abdominal swelling, pain, palpable masses, and hydrothorax have been described in symptomatic cases [8]. The ovarian leiomyoma of our patient was too small to detect during surgery, so it was identified on gross and microscopic examination.

Patients exhibiting ovarian leiomyomas typically range in age between 20 and 65 years. Ovarian leiomyomas are typically unilateral, although bilaterality has been reported in young patients between 16 and 25 [4, 5].

The histogenesis of ovarian leiomyomas is uncertain. They probably arise from smooth muscle cells in the ovarian hilar blood vessels but other possible origins include cells in the ovarian ligament, smooth muscle cells, and undifferentiated germ cells in the ovarian stroma [9]. Endometriotic cysts are also suggested to trigger metaplasia of surrounding stroma into smooth muscle cells [10].

Ovarian leiomyomas should be differentiated from ovarian thecomas, fibromas, and uterine leiomyomas becoming parasites on the ovary. The coexistence of an ovarian leiomyoma with a uterine leiomyoma has been reported by several authors [11, 12]. However, to our knowledge, this is the first case of primary ovarian leiomyoma coexisting with a uterine lipoleiomyoma, two rare entities, in the same individual.

The coexistence of these two rare entities in the same individual may represent a common pathway as a stimulating agent. This case may help and add information to clarify the pathogenesis of these lesions.
